# Inflammatory bowel disease at a young age – implications for achieving upper secondary education

**DOI:** 10.1093/eurpub/ckac131.444

**Published:** 2022-10-25

**Authors:** J Rasmussen, BM Nørgård, RG Nielsen, H Bøggild, N Qvist, RBK Brund, NH Bruun, K Fonager

**Affiliations:** 1 Department of Socialmedicine, Aalborg University Hospital, Aalborg, Denmark; 2 Center for Clinical Epidemiology, Odense University Hospital, Odense, Denmark; 3 Hans Christian Andersen Children’s Hospital, Odense University Hospital, Odense, Denmark; 4 Department of Health Science and Technology, Aalborg University, Aalborg, Denmark; 5 Research Unit for Surgery, Odense University Hospital, Odense, Denmark; 6 Unit of Clinical Biostatistics, Aalborg University Hospital, Aalborg, Denmark; 7 Department of Clinical Medicine, Aalborg University, Aalborg, Denmark

## Abstract

**Background:**

The incidence of inflammatory bowel disease (IBD) among children and adolescence is increasing worldwide. Having a chronic condition at a young age may affect educational achievement and later employment and self-support. The study aims to examine the impact of being diagnosed with IBD before 18 years of age on achieving an upper secondary education before 25 years of age.

**Methods:**

Using the Danish National Patient Register (1980-2018) all patients (born 1970-1994) diagnosed with IBD at a young age (<18 years) were identified. The IBD-patients were matched on age and sex with 10 references without IBD at the index date (date of diagnosis of IBD). The outcome was achieving an upper secondary education using data from Danish Education Registers. The association between IBD diagnosis and achieving an upper secondary education was analyzed using Cox regression with robust variance estimation adjusting for parents’ highest educational level. Furthermore, stratified analyses were performed on parental socioeconomic status (education and income).

**Results:**

We identified 3,178 patients with IBD: Crohn’s disease (CD) n = 1,344, Ulcerative colitis (UC) n = 1,834. Reference n = 28,220. The median age at diagnosis was 15.3 years (IQR: [13.0;16.9]). At the age of 25 74.0% (CI: 71.6-76.4) for CD, 75.8% (CI: 73.8-77.8) for UC, and 69.7% (CI: 69.2-70.3) for references had achieved an upper secondary education. The adjusted Hazard ratio (HR) of achieving an upper secondary education was 1.05 (CI: 1.00 -1.11) for CD and 1.09 (CI: 1.04 -1.15) for UC. When stratifying the IBD-patient with the lowest socioeconomic status performed better than their peers.

**Conclusions:**

Being diagnosed with IBD before 18 years of age did not reduce the chance of achieving an upper secondary education. Patients with low socioeconomic status performed better than their peers, however the study gives no explanation of this.

**Key messages:**

• Children diagnosed with IBD before 18 years of age had at least the same chance of achieving an upper secondary education compared to references.

• IBD patients with low social economic status performed better than their peers.

